# Multiple gene mutations identified in patients infected with influenza A (H7N9) virus

**DOI:** 10.1038/srep25614

**Published:** 2016-05-09

**Authors:** Cuicui Chen, Mingbang Wang, Zhaoqin Zhu, Jieming Qu, Xiuhong Xi, Xinjun Tang, Xiangda Lao, Eric Seeley, Tao Li, Xiaomei Fan, Chunling Du, Qin Wang, Lin Yang, Yunwen Hu, Chunxue Bai, Zhiyong Zhang, Shuihua Lu, Yuanlin Song, Wenhao Zhou

**Affiliations:** 1 Department of Pulmonary Medicine, Zhongshan Hospital, Fudan University, China; 2 Key Laboratory of Birth Defects, Children’s Hospital of Fudan University, Shanghai, China; 3 Division of Neonatology, Children’s Hospital of Fudan University, China; 4 Department of Tuberculosis, Shanghai Public Health Clinical Center, Fudan University, China; 5 Department of Pulmonary Medicine, Shanghai Jiao-Tong University School of Medicine, China; 6 Division of Pulmonary and Critical Care Medicine, University of California, San Francisco, USA; 7 BGI-tech, BGI-Shenzhen, Shenzhen, China; 8 Department of Pulmonary Medicine, Qingpu Central Hospital, Fudan University, China

## Abstract

Influenza A (H7N9) virus induced high mortality since 2013. It is important to elucidate the potential genetic variations that contribute to virus infection susceptibilities. In order to identify genetic mutations that might increase host susceptibility to infection, we performed exon sequencing and validated the SNPS by Sanger sequencing on 18 H7N9 patients. Blood samples were collected from 18 confirmed H7N9 patients. The genomic DNA was captured with the Agilent SureSelect Human All Exon kit, sequenced on the Illumina Hiseq 2000, and the resulting data processed and annotated with Genome analysis Tool. SNPs were verified by independent Sanger sequencing. The DAVID database and the DAPPLE database were used to do bioinformatics analysis. Through exon sequencing and Sanger sequencing, we identified 21 genes that were highly associated with H7N9 influenza infection. Protein-protein interaction analysis showed that direct interactions among genetic products were significantly higher than expected (p = 0.004), and DAVID analysis confirmed the defense-related functions of these genes. Gene mutation profiles of survived and non-survived patients were similar, suggesting some of genes identified in this study may be associated with H7N9 influenza susceptibility. Host specific genetic determinants of disease severity identified by this approach may provide new targets for the treatment of H7N9 influenza.

During the 2013 H7N9 influenza outbreak in southeast China, there were 139 serologically confirmed cases and 48 deaths^1^,^2^. The H7N9 viruses were generated by the subsequent reassortment of H7 viruses with enzootic H9N2 viruses^3^,^4^. H7N9 infection was likely mediated by exposure to poultries because about 55.9% of H7N9 influenza patients had a clearly defined poultry exposure^5^. In addition, closing the poultry trading markets in China was coincided with control of the outbreak. Although many people may have contacted with these poultries, only a minority became sick^6^,^7^, suggesting that there may be genetic determinants of both host susceptibility and severity of infection^8^. The acute onset and rapid progress to severe pneumonia and acute respiratory distress syndrome of H7N9 infection with high mortality highlight the importance of identifying genetic polymorphisms that might predict host response to H7N9 infection. Knowledge of these polymorphisms might help predict both susceptibility to infection and the severity of host response during potential influenza outbreaks in future.

## Material and Methods

### Experimental Design

This study has been approved by Ethics Committee of Fudan University and all experiments were performed in accordance with relevant guidelines and regulations of Ethics Committee of Fudan University. Due to budget limitation, we collected blood samples from 18 H7N9 infected patients during pandemic outbreak period and patients follow up after hospital discharge. We performed exon sequencing on 8 patients of the 12 survivors after H7N9 and verified all the gene mutation in all the 18 blood samples including the non-survivors (Table 1). Control used in house data from BGI-Shenzhen^9^.

### Sample Collection

After informed consents were obtained from all subjects, blood samples were collected from 18 H7N9 infected patients, while exon sequencing was performed on 8 patients. Patients were considered to have H7N9 pneumonia if the following criteria were fulfilled: (1) nasopharyngeal swab positive for H7N9 and; (2) Chest X-ray or CT showing pulmonary infiltrates; (3) clinical symptom of fever and cough. This study was approved by ethical committee of Shanghai Public Health Clinical Center.

### Blood collection and DNA extraction

After consent, 10 ml venous blood was drawn from each patient in an EDTA-containing tube. Samples were immediately centrifuged at 500 g for 10 min and plasma was removed and stored for future measurement. Genomic DNA was extracted from the remaining cell pellet using the SQ Blood DNA Kit II (omegabiotek D0714-250). Briefly, cells were lysed and then cell nuclei and mitochondria were separated by centrifugation. The isolated nuclei were resuspended in XL Buffer (supplied by omegabiotek) which contains chaotropic salt and proteinase to remove contamination. Lastly, genomic DNA was purified by isopropanol precipitation.

### Exome capture, library preparation and sequencing

The isolated genomic DNA from 8 patients was fragmented into DNA strands with lengths of 150 to 200 bp by Covaris technology, and then adapters were ligated to both ends of the resulting fragments. The adapter-ligated templates were purified by the AgencourtAMPure SPRI beads and fragments with the insert size of about 200 bp were excised. Extracted DNA was amplified by ligation-mediated polymerase chain reaction (LM-PCR), purified, and hybridized to Agilent SureSelect Human All Exon (50 M) human exome array for enrichment. Hybridized fragments were bound to strepavidin beads whereas non-hybridized fragments were washed out after 24 h. Captured LM-PCR products were subjected to Agilent 2100 Bio-analyzer to estimate the magnitude of enrichment. Each captured library was then loaded on Hiseq2000 platform, and high-throughput sequencing for each captured library was performed. Raw image files were processed by Illumina base calling Software 1.7 for base calling with default parameters and the sequences of each individual were generated as 90 bp paired-end reads (Table 2).

### Read mapping and variation detection

After removing reads containing sequencing adapters and low-quality reads, high-quality reads were aligned to the NCBI human reference genome (hg19/GRCh37) using BWA (Burrows-Wheeler Aligner, v0.5.9-r16) with default parameters. Low-quality read was defined as more than half of a read was constituted with low quality bases (less than or equal to 5) or a read in which unknown bases were more than 10%. Picard (v1.54) (http://picard.sourceforge.net/) was used to mark duplicates. Subsequently, BAM files (sequence alignment/map format) were compressed to SAM files (the binary files of BAM files). SNPs (Single-nucleotide polymorphism) and InDels (Small insertions/deletions) were detected by module Unified Genotyper of GATK (Genome Analysis Toolkit v1.0.6076). And then ANNOVAR was used to do annotation and classification for SNPs and InDels respectively. Our data have been identified by dbSNP database (http://www.ncbi.nlm.nih.gov/projects/SNP/snp_summary.cgi), 1000 human genomes database (www.1000genomes.org/) and BGI’s inhouse control database. We used BGI’s inhouse control, most controls coming from a Whole Exome Sequencing based study of genetic risk for psoriasis which has been published^9^, and the controls comprised 800 normal people across the whole country. These inhouse control cases were specially used for analysis of rare diseases. Considering only 33 patients had confirmed H7N9 infection in Shanghai, and large population exposed to risk factors, the H7N9 infection was a low possibility case, and could be considered as rare disease, so this control data could be used in this study. We collected 40 genes which were correlated with avian influenza from HuGE Navigator by keyword search with “influenza” and extracted 89 exonic SNVs (single nucleotide variations) (Supplementary Dataset 1) located in 27 genes (Table 3) of the 40 genes from SNPs result.

### Gene mutation verification

The 89 mutations were verified in all the 18 patients by Sanger sequencing. 47 fragments of each patient were amplified from their genomic DNA by PrimeSTAR® HS (Premix) (Takara R040A) to verify the 89 mutated sites. The PCR products were subjected to 1% agarose gel electrophoresis and then purified from the gel by QIAquick Gel Extraction Kit (QIAGEN No. 28706). The purified PCR products were subjected to Sanger sequencing (ABI 3730).

### Bioinformatics analysis

A protein-protein interactions (PPI) network of the resulting genes was constructed using the Disease Association Protein-Protein Link Evaluator (DAPPLE, http://www.broadinstitute.org/mpg/dapple/dapple.php)^8^ with 1000 permutations selected and 2 interacting binding degree as a cutoff. And Database for Annotation, Visualization and Integrated Discovery (DAVID, http://david.abcc.ncifcrf.gov/)^10^, a bioinformatics tool that can identify the biological processes, in which a group of genes are involved, were used for functional annotation.

## Results

### Mutational Analysis of Genes from 18 H7N9 Infected patients

We have admitted 18 H7N9 infected patients around 10 days after disease onset and a series of clinical manifestation, laboratory examinations and prognosis were carried out for the following 15 weeks. 6 of the 18 patients died and we found the increased plasma CRP (Creactive protein), PCT (Procalcitonin) and virus positive days were associated with mortality^11^. After exon sequencing of 8 survivors, 64 exonic SNPs, located in 21 genes, were found to be enriched in the H7N9 patients compared to controls from the NCBI human genome (hg19) (Supplementary Dataset 1 and Table 4). These mutations were found in genes encoding proteins responsible for multiple key host defense mechanisms, including cytokine production, airway epithelium barrier function and pathogen associated molecular pattern signaling pathway, suggesting biological plausibility (Table 2).

### Bioinformatics analysis

**Figure 1:**
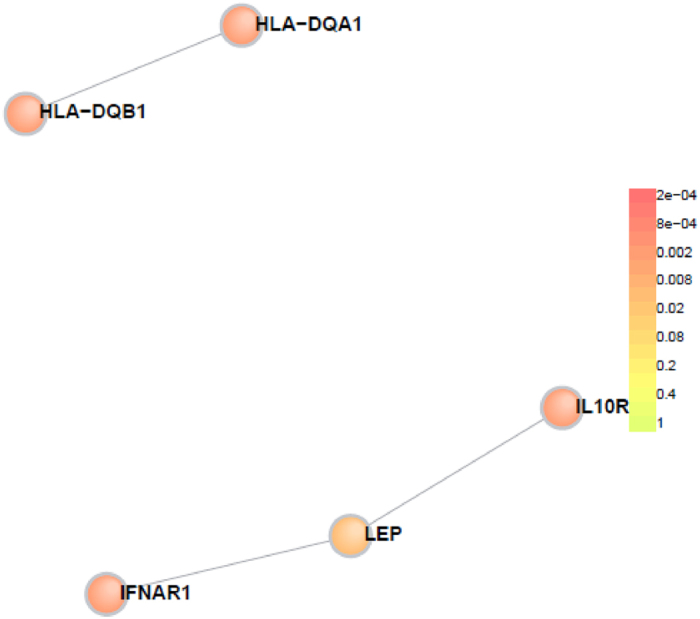
Direct connections among gene products from exome sequencing result. Colours indicate significance of participation in the PPI network.

**Figure 2:**
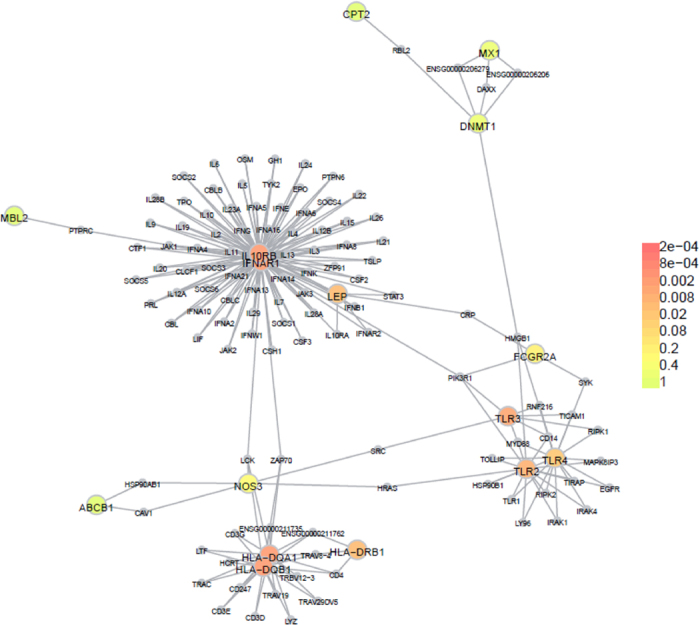
Indirect connections among gene products from exome sequencing result.

The resulting genes with exonic SNPs were uploaded to the online tool DAPPLE for PPI network analysis. The results indicate that the PPI network was statistically significant. There were 5 disease proteins participating in the direct network with 3 direct interactions in total expected direct interactions =  0.347, p =  0.004 (Fig. 1, Table 5). Moreover, there were 13 genes participating in the indirect network under the same condition (Fig. 2).

We further confirmed the functions of these candidate genes using the online tool DAVID. The genes were significantly enriched for defense-related processes such as response to stimulus (p =  1.81 ×  10^−8^), immune response (p =  8.85 ×  10^−7^), immune system process (p =  1.16 ×  10^−6^), response to biotic stimulus (p =  5.48 ×  10^−6^) and modulation by symbiont of host immune response (p =  1.53 ×  10^−5^) (Table 6).

### Gene mutation distribution between different groups

**Figure 3:**
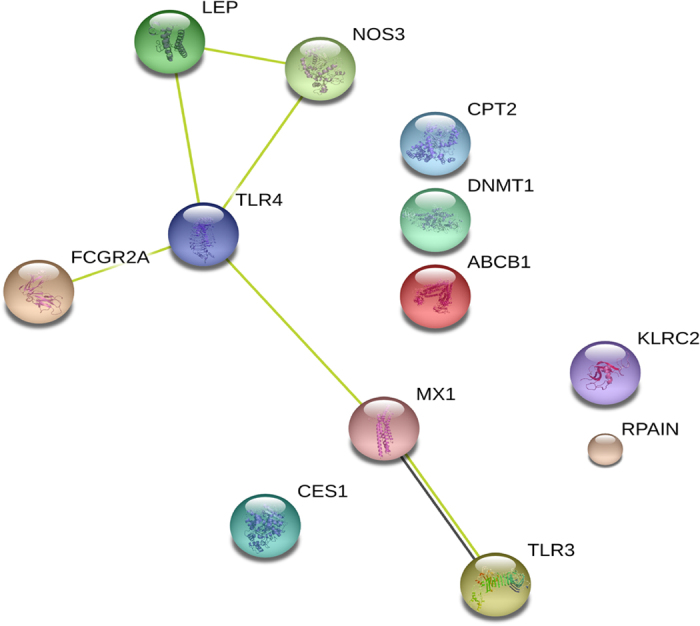
The protein-protein interaction among the 12 genes significantly different between case and control.

Whole exome sequencing was performed on 8 H7N9 patients and 89 exonic SNPs were identified. These SNPs were subjected to Sanger sequencing in all the 18 patients and 64 exonic SNVs were verified. We compared the mutation rate of the case and the inhouse control using the Fisher Exact Test and found significant statistical difference (Supplementary Dataset 1 and Table 4). There were 17 SNVs significantly different between the case and the inhouse control and we have validated 16 of them by Sanger sequencing (Table 7). The 16 validated SNVs were located in 12 genes, and the protein-protein interaction among them (Fig. 3) was consistent with the protein-protein interaction among the 21 genes done before (Fig. 2). It is more likely that both the genes identified from this study that showed statistical difference of mutation frequency and the genes with same mutation rate between patients and controls have participated in the pathogenesis of H7N9 virus infection. We also did Mann-Whitney U test between the first 8 patients and the additional 10 patients and none of P-value was significant (Table 4), which could prove the inhouse control data do not introduce any false signals. Moreover, We compared the mutation rate of death group and survival group and analysis the mutation rate by Mann-Whitney U test and no significant difference was found between the survival and non-survival group (Table 4), suggesting some of genes identified in this study may be associated with H7N9 influenza susceptibility.

## Discussion

The 2013 Chinese H7N9 influenza outbreak lead to an estimated 48 deaths with 33% mortality and significant morbidity in patients who survived the virus. An important observation during the recent H7N9 outbreak in China was the wide variation in host response to infections, with some patients developing only mild upper respiratory tract infections, while other patients developed severe ARDS and died. Although several determinants of the host response to infection have been identified, many important genetic factors that dampen or exacerbate the host response to H7N9 infection likely remain undiscovered. Previous studies suggested that genetic mutations in the protein machinery that comprise key host defense mechanisms could impact outcomes of influenza infection^12^. The differential susceptibilities to influenza A(H7N9) were affected by functional variants of LGALS1 causing the expression variations^13^. The H7N9 influenza outbreak in China provides an unique opportunity to study mutations in this machinery, because many poultry workers were exposed to the virus, yet comparatively few became infected. This may suggest that genetic mutations in host defense mechanisms could be responsible for the selectivity of H7N9 infection. Others have identified genes that are protective during influenza infection, including MX1, NCR1, CCR5, IFITM3 and IL10^14^. Mutations in these genes may lead to increased host susceptibility to infection or to a heightened, and potentially deleterious, host response to infection. We hypothesized that the exome sequencing of these patients may reveal genetic mutations that increased susceptibility to viral infection, and that in the future, these mutations could provide information regarding risk of infection, especially poultry workers or family members of infected patients.

Using a variety of computational genetic techniques, we identified 21 genes that showed a high rate of mutation in patients infected with H7N9 when compared to the general population. Among these genes, some have been identified in prior studies of H7N9 susceptibility genes^14^. For example, Wang *et al.* reported that IFITM3 dysfunction is associated with increased cytokine production during H7N9 infection and is correlated with mortality^14^. IFITM3 (chr11, 320772, A >  G) was reported to be enriched in patients hospitalized due to H1N1/09 infection^15^. Polymorphisms of CPT2, a carnitine palmitoyltransferase 2 protein, were found in patients suffering from influenza-associated encephalopathy; results of overexpression of CPT2 variants *in vitro* suggested that the variants were heat-labile and failed to perform optimally during fever^16^,^17^. Four disease outcome-associated SNPs were identified on chromosomes 17 (RPAIN and C1QBP), chromosome 1 (FCGR2A), and chromosome 3 (unknown gene). C1QBP and GCGR2A play roles in the formation of immune complexes and complement activation, suggesting that the severe disease outcome of H1N1 infection may result from an enhanced host immune response^12^,^16^.

Among the 21 genes we identified, we use the online tool DAPPLE to performed a PPI analysis and found 5 proteins directly participates the PPI network. Those proteins include: LEP, IFNAR1, IL10RB, HLA-DQA1, HLA-DQB1. The PPI analysis suggested significant role of these proteins in influenza infection and may provide target for interventional therapy.

The primary limitation of this study is the relative small sample size. Only 18 patients were enrolled, and confirmation of these findings in subsequent studies will be needed. We are planning to collect more samples for next step sequencing.

## Conclusion

Using comparative genetic analysis in 18 patients with confirmed H7N9 viral infection in China, we identified 21 genetic mutations that occurred at a higher rate in infected patients when compared to the general population. Many of the identified genes are involved in key host defense mechanisms, which gives strong biologic plausibility to the role of these genes in both host susceptibility to infection as well as host immune response related pathology. Further investigations into the function of these genes in host susceptibility may help identify individuals who are at high risk for infection. In addition, translational research into the function of the genes identified in this study may provide new potential therapeutic targets for influenza virus infection.

## Additional Information

**How to cite this article**: Chen, C. *et al.* Multiple gene mutations identified in patients infected with influenza A (H7N9) virus. *Sci. Rep.* **6**, 25614; doi: 10.1038/srep25614 (2016).

## Supplementary Material

Supplementary Information

## Figures and Tables

**Table 1:** The clinical information of the 18 patients.

Number	Survived	Exon sequencing	Poultry exposure	Admitted date	Onset date	Virus postive date
1	N	N	N	2013.4.7	2013.3.17	2013.4.7
2	Y	Y	N	2013.4.13	2013.3.13	2013.4.13
3	Y	N	Y	2013.4.11	2013.4.4	2013.4.10
4	Y	N	N	2013.4.13	2013.4.4	2013.4.13
5	Y	Y	N	2013.4.7	2013.3.30	2013.4.6
6	Y	N	N	2013.4.16	2013.4.11	2013.4.16
7	Y	N	N	2013.4.9	2013.4.4	2013.4.8
8	Y	Y	N	2013.4.10	2013.4.1	2013.4.9
9	N	N	Y	2013.4.6	2013.3.31	2013.4.6
10	N	N	N	2013.4.17	2013.4.10	2013.4.16
11	Y	Y	N	2013.4.13	2013.4.4	2013.4.13
12	N	N	Y	2013.4.18	2013.4.4	2013.4.10
13	N	N	N	2013.4.17	2013.4.11	2013.4.16
14	Y	Y	N	2012.4.9	2013.4.1	2013.4.9
15	N	N	N	2013.4.4	2013.4.2	NA
16	Y	Y	unknown	2013.4.21	2013.4.13	2013.4.20
17	Y	Y	N	2013.4.20	2013.4.13	2013.4.19
18	Y	Y	Y	2013.4.6	2013.4.1	2013.4.6

**Table 2:** Exon sequencing data summary.

Exome capture statistics	Sample							
	P1	P2	P3	P4	P5	P6	P7	P8
Initial bases on target	51543125	51543125	51543125	51543125	51543125	51543125	51543125	51543125
Total effective reads	75899756	79734481	86586748	77653615	88308120	59564257	81497876	101930390
Total effective yield (Mb)	6765.23	7106.16	7730.69	6923.94	7869.24	5310.08	7276.14	9093.9
Average read length (bp)	89.13	89.12	89.28	89.16	89.11	89.15	89.28	89.22
Number of reads uniquely mapped to target	37836108	36730995	37985759	37747936	38384211	35029879	37527575	38836426
Number of reads uniquely mapped to genome	67787246	71481223	77694661	69327742	79613569	52915432	72767207	91803083
Mismatch rate in all effective sequence (%)	0.22	0.23	0.21	0.22	0.21	0.19	0.22	0.22
Average sequencing depth on target (X)	61.64	60.27	62.23	61.71	62.31	57.39	61.78	63.2
Coverage of target region (%)	98.6	99.2	99.1	99.1	98.9	98.6	99.3	99.2
Fraction of target covered > = 20X (%)	79.4	79.9	81.5	89.4	80.1	77.5	81.1	80.6
Fraction of target covered > = 10X (%)	88.8	89.6	90.4	89.7	89.4	87.9	90.2	89.9
Fraction of target covered > = 4X (%)	94.9	95.9	96.2	95.8	95.5	94.6	96.2	96

P, patient. The average sequencing depth on target were all more than 57X in eight samples, Coverage of target region were over 98%.

**Table 3:** 21 virus infection relevant genes identified from exon sequencing.

No	Genes	Full name	Chromosome loci
1	FCGR2A	Fc fragment of IgG	1
2	CPT2	carnitine palmitoyltransferase 2	1
3	TLR2	toll-like receptor 2	4
4	TLR3	toll-like receptor 3	4
5	HLA-DRB1	MHC class II DLA DRB1 beta chain	6
6	HLA-DQA1	MHC class II DLA DRA1 beta chain	6
7	HLA-DQB1	major histocompatibility complex, class II, DQ beta 1	6
8	NOS3	nitric oxide synthase 3	7
9	LEP	leptin	7
10	ABCB1	ATP-binding cassette, sub-family B	7
11	TLR4	toll-like receptor 4	9
12	IFNAR1	interferon (alpha, beta and omega) receptor 1	21
13	IL10RB	interleukin 10 receptor	21
14	MBL2	mannose-binding lectin (protein C) 2	10
15	ZNF365	zinc finger protein 365	10
16	IFITM3	interferon induced transmembrane protein 3	11
17	KLRC2	killer cell lectin-like receptor subfamily C, member 2	12
18	CES1	carboxylesterase 1	16
19	RPAIN	RPA interacting protein	17
20	DNMT1	DNA (cytosine-5-)-methyltransferase 1	19
21	MX1	MX dynamin-like GTPase 1	21

**Table 4:** The 64 validated exonic SNVs and their distribution between different groups.

Gene Information					Comparison between survived and deceased			Comparison between the first 8 patients and the additional 10 pateints			Comparison between case and control			
Gene	Chr	Pos	Ref	Alt	Mutation in 12 survivors	Mutation in 6 decedents	P-value	Mutation in first 8 patients	Mutation in the other 10 pateints	P-value	1000 Genome	Inhouse control	dbSNP	P-value between case and inhouse control
CPT2	chr1	53676401	T	G	5	2	1	4	3	1	0.06	0.2104	rs2229291	0.352872195
CPT2	chr1	53676448	G	A	12	4	0.4	8	8	0.7	0.5	0.669	rs1799821	0.107976012
CPT2	chr1	53679028	G	A	1	0	0.7	1	0	0.7		0.0008		0.019710021
CPT2	chr1	53679229	A	G	2	3	1	4	1	1	0.13	0.1368	rs1799822	1
FCGR2A	chr1	161479745	A	G	6	3	0.7	3	6	0.4	0.43	0.4777	rs1801274	0.023221855
IFITM3	chr11	320649	G	A	0	2	0.2	0	2	0.2		0.2488	rs11553885	0.140015157
IFITM3	chr11	320772	A	G	12	5	0.1	8	9	0.2	0.21	0.3101	rs12252	0.110209014
RPAIN	chr17	5326145	C	G	12	5	0.4	8	9	0.4	0.43	0.6017	rs12761	0.036500478
TLR3	chr4	187004074	C	T	9	2	0.1	6	5	0.4	0.25	0.3324	rs3775291	0.028998842
TLR3	chr4	187004217	C	T	5	4	1	3	9	0.1	0.28	0.3702	rs3775290	0.192232214
TLR2	chr4	154624656	T	C	5	5	0.7	5	5	0.7	0.43	0.4474	rs3804099	0.622004107
TLR2	chr4	154625259	A	G	1	0	0.7	1	0	0.7	0.0023	0.0239	rs144038898	0.32478413
TLR2	chr4	154625409	T	C	4	5	1	4	5	1	0.12	0.4378	rs3804100	0.801147802
HLA-DQA1	chr6	32605284	G	A	1	0	0.2	1	0	0.2	0.06	0.0766	rs12722039	0.350824196
HLA-DQA1	chr6	32605309	A	G	1	0	0.2	1	0	0.2	0.06	0.0622	rs12722042	0.263992352
HLA-DQA1	chr6	32609094	C	T	2	5	0.4	2	5	0.4	0.35	0.2225	rs1129737	0.54610042
HLA-DQA1	chr6	32609147	A	T	0	2	1	0	2	1	0.19	0.2679	rs12722051	0.262995374
HLA-DQA1	chr6	32609173	C	G	2	5	0.2	2	5	0.2	0.4	0.2656	rs10093	0.264047871
HLA-DQA1	chr6	32609195	G	A	1	5	1	1	5	1	0.09	0.1675	rs36219699	1
HLA-DQA1	chr6	32609813	T	C	5	8	0.4	5	8	0.4	0.66	0.2919	rs707951	0.174782886
HLA-DQA1	chr6	32610436	T	C	6	8	0.4	6	8	0.4	0.61	0.2679	rs1048372	0.262995374
HLA-DQA1	chr6	32610535	A	C	6	8	0.4	6	8	0.4	0.57	0.2703	rs1130116	0.262188523
HLA-DQB1	chr6	32629129	T	C	1	2	0.4	1	2	0.4	0.19	0.2416	rs1130432	0.384909932
HLA-DQB1	chr6	32629155	C	A	1	2	0.4	1	2	0.4	0.23	0.2153	rs17412886	0.545857944
HLA-DQB1	chr6	32629847	A	G	7	9	0.7	7	9	0.7	0.8	0.3541	rs1049133	0.444388806
HLA-DQB1	chr6	32629859	A	G	6	9	0.7	6	9	0.7	0.65	0.3421	rs1049130	0.59863218
HLA-DQB1	chr6	32629868	A	G	2	5	1	2	5	0.1	0.18	0.134	rs1049088	1
HLA-DQB1	chr6	32629889	G	A	5	7	0.4	5	7	0.4	0.44	0.2392	rs1049087	0.385697708
HLA-DQB1	chr6	32629904	A	G	5	6	0.4	5	6	0.4	0.6	0.2656	rs1049086	0.264047871
HLA-DQB1	chr6	32629936	C	T	1	2	0.4	1	2	0.4	0.21	0.2057	rs1049107	0.548651412
HLA-DQB1	chr6	32629963	C	T	1	2	0.4	1	2	0.4	0.2	0.177	rs1049100	0.752136224
HLA-DQB1	chr6	32634313	C	G	1	2	0.4	1	2	0.4	0.21	0.0885	rs1049059	0.646223667
HLA-DQB1	chr6	32634369	C	A	1	2	0.4	1	2	0.4	0.21	0.0981	rs1049056	0.664995324
HLA-DRB1	chr6	32549596	T	C	8	4	0.4	8	4	0.4		0.311	rs111823233	0.171375278
NOS3	chr7	150695726	T	C	8	9	0.7	8	9	0.7	0.78	0.6699	rs1549758	0.182947056
NOS3	chr7	150696111	T	G	8	9	0.4	8	9	0.4	0.8	0.8732	rs1799983	1
NOS3	chr7	150704250	C	G	4	1	1	4	1	1	0.42	0.5096	rs2566514	0.04531093
ABCB1	chr7	87138645	A	G	7	8	0.7	7	8	0.7	0.6	0.6507	rs1045642	0.110998732
ABCB1	chr7	87160618	A	T or C	7	5	0.7	7	5	0.7		0.1244	rs2032582	0.709435825
ABCB1	chr7	87179601	A	G	3	4	0.7	3	4	0.7	0.58	0.4617	rs1128503	0.040826503
LEP	chr7	127892124	A	G	1	0	0.7	1	0	0.7	0.0005	0.0024	rs148407750	0.039055387
TLR4	chr9	120470894	C	G	1	0	0.7	1	0	0.7		–		0.00990099
MBL2	chr10	54528266	G	C	8	9	1	8	9	1	0.77	0.6388	rs930507	0.194023134
MBL2	chr10	54531235	C	T	3	3	1	3	3	1	0.12	0.2057	rs1800450	1
ZNF365	chr10	64159333	G	T	7	7	0.7	7	7	0.7	0.49	0.7967	rs3758490	0.11468297
ZNF365	chr10	64415184	A	G	8	9	1	8	9	1	0.85	0.9115	rs7076156	1
ZNF365	chr10	64416220	C	T	0	1	0.7	0	1	0.7	0.02	0.0359	rs76895268	0.444340031
KLRC2	chr12	10587111	A	G	2	9	1	2	9	1	0.76	0.4234	rs1141715	0.003533467
KLRC2	chr12	10588530	C	G	6	5	1	6	5	1	0.26	0.4785	rs34195537	0.004729536
CES1	chr16	55844509	T	C	2	0	0.7	2	0	0.7		–		0.00990099
CES1	chr16	55853545	C	A	0	4	0.7	0	4	0.7	0.04	0.0407	rs115629050	0.488479286
CES1	chr16	55855361	G	T	1	0	0.7	1	0	0.7	0.04	0.0311	rs2307227	0.396649049
DNMT1	chr19	10251572	G	C	1	0	0.7	1	0	0.7	0.0037	0.0072	rs144675407	0.112914886
DNMT1	chr19	10265312	T	C	8	9	0.4	8	9	0.4	0.99	0.7297	rs721186	0.00938611
DNMT1	chr19	10265333	A	G	1	0	0.7	1	0	0.7		–		0.00990099
DNMT1	chr19	10265372	C	T	1	0	0.7	1	0	0.7	0.0005	–	rs140376680	0.00990099
DNMT1	chr19	10267077	T	C	8	7	0.4	8	7	0.4	0.54	0.6388	rs2228611	0.194023134
DNMT1	chr19	10291181	T	C	0	2	0.2	0	2	0.2	0.06	0.3756	rs16999593	0.437380251
IFNAR1	chr21	34715699	G	C	4	7	0.1	4	7	0.1	0.21	0.5239	rs2257167	0.129919992
MX1	chr21	42812891	C	T	1	2	1	1	2	1	0.36	0.177	rs467960	0.332738289
MX1	chr21	42817930	G	A	1	1	1	1	1	1	0.43	0.4474	rs469390	0.001564732
MX1	chr21	42821113	T	C	4	2	0.4	4	2	0.4	0.35	0.3278	rs2070229	0.422944246
MX1	chr21	42824661	A	G	4	4	0.7	4	4	0.7	0.27	0.5215	rs1050008	0.042066821
IL10RB	chr21	34640788	A	G	8	1	0.1	8	1	0.1	0.35	0.7392	rs2834167	1

**Table 5:** The PPI network statistics.

PARAMETER	OBSERVED	EXPECTED	P_VALUE
Direct Edges Count	3	0.347	0.003996004
Seed Direct Degrees Mean	1.2	0.3024	0.014985015
Seed Indirect Degrees Mean	17.25	3.732515889	0.000999001
CI Degrees Mean	2.096491	2.067364797	0.265734266

**Table 6:** Top 10 go term analysis results.

Term	Count	percentage	P-Value	Genes
GO:0050896:response to stimulus	17	80.95238095	1.81E-08	HLA-DQB1, MBL2, KLRC2, CES1, HLA-DRB1, IFITM3, TLR2, TLR3, ABCB1, TLR4, HLA-DQA1, IFNAR1, LEP, RPAIN, IL10RB, NOS3, MX1
GO:0006955:immune response	9	42.85714286	8.85E-07	HLA-DQB1, MBL2, HLA-DRB1, IL10RB, IFITM3, TLR2, TLR3, TLR4, HLA-DQA1
GO:0002376:immune system process	10	47.61904762	1.16E-06	HLA-DQB1, MBL2, HLA-DRB1, IL10RB, IFITM3, TLR2, TLR3, TLR4, HLA-DQA1, IFNAR1
GO:0009607:response to biotic stimulus	7	33.33333333	5.48E-06	IFITM3, TLR2, TLR3, NOS3, TLR4, MX1, IFNAR1
GO:0052553:modulation by symbiont of host immune response	3	14.28571429	1.53E-05	TLR2, TLR3, TLR4
GO:0052556:positive regulation by symbiont of host immune response	3	14.28571429	1.53E-05	TLR2, TLR3, TLR4
GO:0052166:positive regulation by symbiont of host innate immunity	3	14.28571429	1.53E-05	TLR2, TLR3, TLR4
GO:0052306:modulation by organism of innate immunity in other organism during symbiotic interaction	3	14.28571429	1.53E-05	TLR2, TLR3, TLR4
GO:0052305:positive regulation by organism of innate immunity in other organism during symbiotic interaction	3	14.28571429	1.53E-05	TLR2, TLR3, TLR4
GO:0052555:positive regulation by organism of immune response of other organism during symbiotic interaction	3	14.28571429	1.53E-05	TLR2, TLR3, TLR4

**Table 7:** The 17 SNVs significantly different between case and control.

Gene	Chr	Pos	Ref	Obs	caseRef	caseAlt	controlRef	controlAlt	FisherExactTestPvalue	Sanger sequencing validated
PPARG	chr3	12475632	G	A	15	1	1600	0	0.00990099	No
CPT2	chr1	53679028	G	A	15	1	1599	1	0.019710021	Yes
FCGR2A	chr1	161479745	A	G	13	3	836	764	0.023221855	Yes
RPAIN	chr17	5326145	C	G	2	14	638	962	0.036500478	Yes
TLR3	chr4	187004074	C	T	6	10	1069	531	0.028998842	Yes
NOS3	chr7	150704250	C	G	12	4	785	815	0.04531093	Yes
ABCB1	chr7	87179601	A	G	13	3	862	738	0.040826503	Yes
LEP	chr7	127892124	A	G	15	1	1597	3	0.039055387	Yes
TLR4	chr9	120470894	C	G	15	1	1600	0	0.00990099	Yes
KLRC2	chr12	10587111	A	G	3	13	923	677	0.003533467	Yes
	chr12	10588530	C	G	14	2	835	765	0.004729536	Yes
CES1	chr16	55844509	T	C	15	1	1600	0	0.00990099	Yes
DNMT1	chr19	10265312	T	C	0	16	433	1167	0.00938611	Yes
	chr19	10265333	A	G	15	1	1600	0	0.00990099	Yes
	chr19	10265372	C	T	15	1	1600	0	0.00990099	Yes
MX1	chr21	42817930	G	A	15	1	885	715	0.001564732	Yes
	chr21	42824661	A	G	12	4	766	834	0.042066821	Yes
