# Supplementary material for: Multiple gene mutations identified in patients infected with influenza A (H7N9) virus
Source: Sci Rep. 2016 May 9;6:25614. doi: 10.1038/srep25614 (PMC4860572; doi:10.1038/srep25614)
Supplement: Supplementary Information [file srep25614-s1.pdf]

Multiple gene mutations identified in patients infected with influenza A (H7N9) virus

[1] Cuicui Chen*, [2]Mingbang Wang*,[3]Zhaoqin Zhu*, [4]Jieming Qu*, [1]Xiangda Lao*, [5]Eric Seeley*, [3]Xiuhong Xi*, [1]Xinjun Tang*,[3] Tao Li, [6]Xiaomei Fan, [7]Chunling Du, [1]Qin Wang, [2]Lin Yang, [3]Yunwen Hu, [1]Chunxue Bai, [4]Zhiyong Zhang, [3]Shuihua Lu[#], [1,3,7]Yuanlin Song[#], [2]Wenhao Zhou[#]

[1]Department of Pulmonary Medicine, Zhongshan Hospital, Fudan University;

[2] Key Laboratory of Birth Defects, Children's Hospital of Fudan University, Shanghai, China; Division of Neonatology, Children's Hospital of Fudan University, China;

[3]Department of Tuberculosis, Shanghai Public Health Clinical Center ,Fudan University;

[4]Department of Pulmonary Medicine, Shanghai Jiao-Tong University School of Medicine;

[5]Division of Pulmonary and Critical Care Medicine, University of California, San Francisco;

[6]BGI-tech, BGI-Shenzhen, Shenzhen, China.

[7]Department of Pulmonary Medicine, Qingpu Central Hospital, Fudan University.

*Those authors contributed equally to this work.

# correspondence to:

Shuihua Lu, M.D.

Department of Tuberculosis; Shanghai Public Health Clinical Center

Email: lushuihua66@126.com

Yuanlin Song, M.D.

Department of Pulmonary Medicine, Zhongshan Hospital, Fudan University

180 Fenglin Road, Shanghai, 200032, PRChina

Tel: 86-21-64041990-2963

Email: ylsong70@163.com

Wenhao Zhou, M.D., Ph.D.

Key Laboratory of Birth Defects, Children's Hospital of Fudan University, Shanghai, China;

Division of Neonatology, Children's Hospital of Fudan University, China

Email: zwhchfu@126.com

Table S1. The 89 exonic SNVs and their mutation rate in various groups.

Gene	Chr	Pos	Ref	Alt	Fun	Exonic Func	1000 Genome	Inhouse	dbSNP	P1	P2	P3	P4	P5	P6	P7	P8	case Ref	case Alt	con Ref	con Alt	Fisher Exact Test P-value
CPT2	chr1	53679028	G	A	exonic	nonsynonymous SNV		0.0008		-	-	-	-	-	het:32,36	-	-	15	1	1599	1	0.019710021
LEP	chr7	127892124	A	G	exonic	nonsynonymous SNV	0.0005	0.0024	rs148407750	-	-	-	-	-	het:67,61	-	-	15	1	1597	3	0.039055387
DNMT1	chr19	10251572	G	C	exonic	synonymous SNV	0.0037	0.0072	rs144675407	-	-	-	-	-	-	-	het:29,20	15	1	1589	11	0.112914886
IL1B	chr2	113590390	G	A	exonic	synonymous SNV	0.15	0.0144	rs1143634	-	-	-	het:30,24	-	-	-	-	15	1	1577	23	0.213791053
TLR2	chr4	154625259	A	G	exonic	synonymous SNV	0.0023	0.0239	rs144038898	-	-	-	-	het:30,42	-	-	-	15	1	1562	38	0.32478413
CES1	chr16	55855361	G	T	exonic	nonsynonymous SNV	0.04	0.0311	rs2307227	-	-	-	-	-	het:25,13	-	-	15	1	1551	49	0.396649049
IL1B	chr2	113591114	G	A	exonic	synonymous	0.01	0.0335	rs140794289	-	-	-	-	-	-	het:20,13	-	15	1	1547	53	0.420954673

						SNV																
ZNF365	chr10	64416220	C	T	exonic	synonymous SNV	0.02	0.0359	rs76895268	-	het:16,20	-	-	-	-	-	-	15	1	1543	57	0.444340031
CES1	chr16	55853545	C	A	exonic	nonsynonymous SNV	0.04	0.0407	rs115629050	-	-	-	-	-	het:101,46	-	-	15	1	1535	65	0.488479286
HLA-DQB1	chr6	32632801	G	A	exonic	synonymous SNV	0.05	0.0526	rs3204373	-	-	hom:0,7	-	-	-	-	-	14	2	1516	84	0.207841802
HLA-DQB1	chr6	32632833	A	G	exonic	nonsynonymous SNV	0.05	0.0526	rs12722107	-	-	hom:0,6	-	-	-	-	-	14	2	1516	84	0.207841802
HLA-DQA1	chr6	32605309	A	G	exonic	nonsynonymous SNV	0.06	0.0622	rs12722042	-	-	-	hom:0,12	-	-	-	-	14	2	1501	99	0.263992352
PPARG	chr3	12458274	C	G	exonic	synonymous SNV	0.01	0.067	rs13306747	-	-	-	-	-	het:24,22	-	-	15	1	1493	107	1
HLA-DQB1	chr6	32632642	C	T	exonic	synonymous SNV	0.06	0.067	rs3204379	-	-	hom:0,11	-	-	-	-	-	14	2	1493	107	0.294264656
HLA-DQA1	chr6	32605284	G	A	exonic	nonsynonymous SNV	0.06	0.0766	rs12722039	-	-	-	hom:0,14	-	-	-	-	14	2	1478	122	0.350824196
HLA-	chr	3263	T	C	exo	nonsyn	0.21	0.0789	rs1130	-	-	-	hom:	-	-	-	-	14	2	1474	126	0.36575

DQB1	6	2647			nic	onymous SNV			390				0,7									7012
HLA-DQB1	chr6	32632688	T	A	exonic	nonsynonymous SNV	0.2	0.0789	rs1071637	-	-	-	hom:0,10	-	-	-	-	14	2	1474	126	0.365757012
HLA-DQB1	chr6	32634313	C	G	exonic	nonsynonymous SNV	0.21	0.0885	rs1049059	-	-	-	hom:0,16	-	-	-	-	14	2	1459	141	0.646223667
LEPR	chr1	66067109	T	C	exonic	synonymous SNV	0.19	0.0957	rs1805134	-	-	-	het:33,29	-	-	-	-	15	1	1447	153	1
HLA-DQB1	chr6	32634369	C	A	exonic	nonsynonymous SNV	0.21	0.0981	rs1049056	-	-	-	hom:0,12	-	-	-	-	14	2	1444	156	0.664995324
HLA-DQB1	chr6	32632820	C	G	exonic	nonsynonymous SNV	0.22	0.11	rs1130375	-	-	hom:0,8	-	-	-	-	-	14	2	1424	176	0.693524126
HLA-DQB1	chr6	32632770	A	G	exonic	nonsynonymous SNV	0.28	0.1124	rs1049069	-	-	-	hom:0,6	-	-	-	-	14	2	1421	179	0.698016409
CES1	chr16	55862762	C	G	exonic	synonymous SNV		0.1124	rs3826194	-	-	-	-	-	het:28,7	-	-	15	1	1421	179	1
ABCB1	chr7	87160618	A	T	exonic	nonsynonymous SNV		0.1244	rs2032582	-	-	-	-	-	-	-	het:47,47	15	1	1401	199	0.709435825

HLA-DQB1	chr 6	32629868	A	G	exonic	synonymous SNV	0.18	0.134	rs1049088	-	-	-	hom:0,23	-	-	-	-	14	2	1386	214	1
CRP	chr 1	159683438	C	G	exonic	synonymous SNV	0.04	0.1364	rs1800947	-	-	-	-	het:54,45	-	het:47,52	-	14	2	1382	218	1
CPT2	chr 1	53679229	A	G	exonic	nonsynonymous SNV	0.13	0.1368	rs1799822	-	het:5,19	-	-	het:24,22	-	-	-	14	2	1382	218	1
HLA-DQB1	chr 6	32632745	G	A	exonic	nonsynonymous SNV	0.33	0.1388	rs1063318	-	-	-	hom:0,11	-	-	-	-	14	2	1378	222	1
HLA-DQA1	chr 6	32609195	G	A	exonic	nonsynonymous SNV	0.09	0.1675	rs36219699	-	-	hom:0,30	-	-	-	-	-	14	2	1332	268	1
IL1A	chr 2	113537223	C	A	exonic	nonsynonymous SNV	0.2	0.1675	rs17561	-	-	-	het:30,24	het:14,21	-	het:20,30	-	13	3	1332	268	0.740919053
HLA-DQB1	chr 6	32629963	C	T	exonic	nonsynonymous SNV	0.2	0.177	rs1049100	-	-	-	hom:0,21	-	-	-	-	14	2	1317	283	0.752136224
MX1	chr 21	42812891	C	T	exonic	synonymous SNV	0.36	0.177	rs467960	-	-	het:29,36	-	-	-	-	-	15	1	1317	283	0.332738289
CES1	chr 16	55862691	G	A	exonic	nonsynonymo	0.38	0.1794	rs62028647	-	-	-	-	-	het:28,7	-	-	15	1	1313	287	0.332096782

						us SNV																
HLA-DQB1	chr 6	32629936	C	T	exonic	nonsynonymous SNV	0.21	0.2057	rs1049107	-	-	-	hom:0,27	-	-	-	-	14	2	1271	329	0.548651412
MBL2	chr 10	54531235	C	T	exonic	nonsynonymous SNV	0.12	0.2057	rs1800450	het:23,18	-	het:21,13	het:27,23	-	-	-	-	13	3	1271	329	1
CPT2	chr 1	53676401	T	G	exonic	nonsynonymous SNV	0.06	0.2104	rs2229291	het:18,20	-	het:19,22	hom:0,42	-	-	-	het:31,42	11	5	1264	336	0.352872195
HLA-DQB1	chr 6	32629155	C	A	exonic	synonymous SNV	0.23	0.2153	rs17412886	-	-	-	hom:0,24	-	-	-	-	14	2	1256	344	0.545857944
HLA-DQA1	chr 6	32609094	C	T	exonic	synonymous SNV	0.35	0.2225	rs1129737	-	-	hom:0,45	-	-	-	-	-	14	2	1244	356	0.54610042
HLA-DQB1	chr 6	32629889	G	A	exonic	synonymous SNV	0.44	0.2392	rs1049087	-	-	hom:0,6	-	-	-	-	-	14	2	1218	382	0.385697708
HLA-DQB1	chr 6	32632650	C	T	exonic	nonsynonymous SNV	0.56	0.2392	rs1130386	-	-	hom:0,11	-	-	-	-	-	14	2	1218	382	0.385697708
HLA-DQB1	chr 6	32629129	T	C	exonic	nonsynonymous SNV	0.19	0.2416	rs1130432	-	-	-	hom:0,26	-	-	-	-	14	2	1214	386	0.384909932
IFIT	chr1	3206	G	A	exo	synony		0.2488	rs1155	-	-	het:6	-	-	-	-	-	15	1	1202	398	0.14001

M3	1	49			nic	mous SNV			3885			7,14										5157
HLA-DQA1	chr6	32609173	C	G	exonic	nonsynonymous SNV	0.4	0.2656	rs10093	-	-	-	hom: 0,36	-	-	-	-	14	2	1176	424	0.264047871
HLA-DQB1	chr6	32629904	A	G	exonic	synonymous SNV	0.6	0.2656	rs1049086	-	-	hom: 0,7	-	-	-	-	-	14	2	1176	424	0.264047871
HLA-DQA1	chr6	32609147	A	T	exonic	nonsynonymous SNV	0.19	0.2679	rs12722051	-	-	hom: 0,36	-	-	-	-	-	14	2	1172	428	0.262995374
HLA-DQA1	chr6	32610436	T	C	exonic	synonymous SNV	0.61	0.2679	rs1048372	-	-	-	hom: 0,32	-	-	-	-	14	2	1172	428	0.262995374
HLA-DQA1	chr6	32610535	A	C	exonic	synonymous SNV	0.57	0.2703	rs1130116	-	-	hom: 0,52	-	-	-	-	-	14	2	1168	432	0.262188523
HLA-DQA1	chr6	32609813	T	C	exonic	synonymous SNV	0.66	0.2919	rs707951	-	-	hom: 0,25	-	-	-	-	-	14	2	1133	467	0.174782886
PPARG	chr3	12475557	C	T	exonic	synonymous SNV	0.12	0.3086	rs3856806	het:39,31	-	-	-	het:25,33	-	-	het:33,22	13	3	1107	493	0.417143794
IFITM3	chr11	320772	A	G	exonic	synonymous SNV	0.21	0.3101	rs12252	-	hom: 0,119	-	hom: 0,114	-	hom: 0,99	het:57,60	het:65,65	8	8	1104	496	0.110209014

HLA-DRB1	chr 6	32549596	T	C	exonic	nonsynonymous SNV		0.311	rs111823233	-	-	-	hom: 0,7	-	-	-	-	14	2	1103	497	0.171375278
MX1	chr 21	42821113	T	C	exonic	synonymous SNV	0.35	0.3278	rs2070229	hom: 0,83	hom: 1,80	-	-	hom: 0,78	-	het:53,41	-	9	7	1076	524	0.422944246
TLR3	chr 4	187004074	C	T	exonic	nonsynonymous SNV	0.25	0.3324	rs3775291	hom: 0,68	het:32,20	-	hom: 0,43	hom: 1,44	-	hom: 0,50	het:27,31	6	10	1069	531	0.028998842
HLA-DQB1	chr 6	32629859	A	G	exonic	synonymous SNV	0.65	0.3421	rs1049130	-	-	hom: 0,4	hom: 0,22	-	-	-	-	12	4	1053	547	0.59863218
HLA-DQB1	chr 6	32629847	A	G	exonic	synonymous SNV	0.8	0.3541	rs1049133	-	-	hom: 0,4	hom: 0,17	-	-	-	-	12	4	1034	566	0.444388806
GHRL	chr 3	10331457	G	T	exonic	nonsynonymous SNV	0.09	0.3612	rs696217	-	hom: 0,76	-	-	-	-	het:51,52	-	13	3	1023	577	0.194240734
TLR3	chr 4	187004217	C	T	exonic	synonymous SNV	0.28	0.3702	rs3775290	-	-	het:38,33	-	-	het:31,28	-	het:33,44	13	3	1008	592	0.192232214
DNMT1	chr 19	10291181	T	C	exonic	nonsynonymous SNV	0.06	0.3756	rs16999593	het:54,43	-	het:53,30	-	het:52,53	-	het:45,55	-	12	4	1000	600	0.437380251
HLA-DQA1	chr 6	32610461	A	G	exonic	nonsynonymo	0.78	0.4115	rs9260	-	-	hom: 0,41	hom: 0,31	-	-	-	-	12	4	942	658	0.21390613

						us SNV																
KLRC2	chr12	10587111	A	G	exonic	nonsynonymous SNV	0.76	0.4234	rs1141715	hom:0,241	hom:0,209	het:59,118	hom:0,162	hom:0,245	het:56,194	het:61,115	hom:0,186	3	13	923	677	0.003533467
TLR2	chr4	154625409	T	C	exonic	synonymous SNV	0.12	0.4378	rs3804100	-	-	hom:0,93	-	het:44,40	het:47,46	het:59,44	het:39,55	10	6	900	700	0.801147802
MX1	chr21	42817930	G	A	exonic	nonsynonymous SNV	0.43	0.4474	rs469390	-	-	het:23,32	-	-	-	-	-	15	1	885	715	0.001564732
TLR2	chr4	154624656	T	C	exonic	synonymous SNV	0.43	0.4474	rs3804099	-	-	hom:0,69	-	het:37,41	het:30,21	het:32,46	het:35,34	10	6	885	715	0.622004107
ABCB1	chr7	87179601	A	G	exonic	synonymous SNV	0.58	0.4617	rs1128503	-	-	-	het:29,28	-	-	het:23,23	het:29,21	13	3	862	738	0.040826503
FCGR2A	chr1	161479745	A	G	exonic	nonsynonymous SNV	0.43	0.4777	rs1801274	-	het:45,43	het:30,37	-	-	-	het:30,30	-	13	3	836	764	0.023221855
KLRC2	chr12	10588530	C	G	exonic	nonsynonymous SNV	0.26	0.4785	rs34195537	het:118,66	-	-	-	-	het:168,33	-	-	14	2	835	765	0.004729536
NOS3	chr7	150704250	C	G	exonic	synonymous SNV	0.42	0.5096	rs2566514	-	-	het:21,21	het:24,33	het:32,20	-	-	het:21,22	12	4	785	815	0.04531093
ABC	chr	8716	A	C	exo	nonsyn	0.66	0.512	rs2032	het:4	het:5	het:3	het:5	het:4	-	het:4	-	10	6	781	819	0.32130

B1	7	0618			nic	onymous SNV			582	6,44	4,45	8,40	3,42	5,39		5,41						5592
MX1	chr21	42824661	A	G	exonic	synonymous SNV	0.27	0.5215	rs1050008	-	-	het:89,85	het:77,71	-	het:67,64	het:78,75	-	12	4	766	834	0.042066821
IFNAR1	chr21	34715699	G	C	exonic	nonsynonymous SNV	0.21	0.5239	rs2257167	-	-	hom:0,80	het:49,45	-	het:57,54	-	het:54,59	11	5	762	838	0.129919992
DNMT1	chr19	10273372	T	C	exonic	nonsynonymous SNV	0.18	0.5383	rs2228612	het:19,21	-	het:28,24	het:20,22	het:22,20	-	het:21,31	-	11	5	739	861	0.08161176
RPAIN	chr17	5326145	C	G	exonic	nonsynonymous SNV	0.43	0.6017	rs12761	hom:0,24	hom:0,24	hom:0,27	het:13,10	hom:0,34	hom:0,20	hom:0,25	het:23,12	2	14	638	962	0.036500478
LEPR	chr1	66102257	G	A	exonic	synonymous SNV	0.54	0.61	rs1805096	hom:0,104	het:62,34	het:53,38	hom:0,105	hom:0,104	het:41,38	hom:0,122	hom:0,112	3	13	624	976	0.123833455
DNMT1	chr19	10267077	T	C	exonic	synonymous SNV	0.54	0.6388	rs2228611	het:28,27	hom:0,70	het:28,30	het:32,34	hom:0,58	hom:0,42	hom:0,62	hom:0,47	3	13	578	1022	0.194023134
MBL2	chr10	54528266	G	C	exonic	synonymous SNV	0.77	0.6388	rs930507	hom:0,85	hom:0,88	het:44,41	hom:0,75	het:45,55	hom:0,60	hom:0,97	het:42,47	3	13	578	1022	0.194023134
ABCB1	chr7	87138645	A	G	exonic	synonymous SNV	0.6	0.6507	rs1045642	het:58,43	het:35,39	het:49,39	het:48,52	het:37,30	-	het:42,47	het:36,43	9	7	559	1041	0.110998732

CPT2	chr1	53676448	G	A	exonic	nonsynonymous SNV	0.5	0.669	rs1799821	hom:0,38	hom:0,35	het:26,22	hom:0,42	het:11,21	hom:0,39	hom:0,49	hom:0,54	2	14	530	1070	0.107976012
NOS3	chr7	150695726	T	C	exonic	synonymous SNV	0.78	0.6699	rs1549758	hom:0,8	-	-	-	hom:0,10	-	hom:0,7	hom:0,7	8	8	529	1071	0.182947056
LEPR	chr1	66058513	A	G	exonic	nonsynonymous SNV	0.59	0.6986	rs1137101	hom:0,53	hom:0,46	het:35,25	hom:0,60	hom:0,42	het:25,24	hom:0,50	hom:0,48	2	14	483	1117	0.171547862
DNMT1	chr19	10265312	T	C	exonic	synonymous SNV	0.99	0.7297	rs721186	hom:0,39	hom:0,32	hom:0,38	hom:0,32	hom:0,34	hom:0,23	hom:0,47	hom:0,38	0	16	433	1167	0.00938611
LEPR	chr1	66036441	A	G	exonic	nonsynonymous SNV	0.39	0.7344	rs1137100	hom:0,63	hom:0,57	het:18,34	het:42,29	hom:0,82	het:13,35	hom:0,47	het:27,44	4	12	425	1175	1
IL10RB	chr21	34640788	A	G	exonic	nonsynonymous SNV	0.35	0.7392	rs2834167	hom:0,37	hom:0,59	het:33,18	het:26,20	het:19,24	hom:0,46	hom:0,48	het:20,36	4	12	418	1182	1
ZNF365	chr10	64159333	G	T	exonic	nonsynonymous SNV	0.49	0.7967	rs3758490	het:59,61	het:69,57	het:88,56	het:56,60	hom:0,119	hom:0,98	hom:0,102	-	6	10	326	1274	0.11468297
NOS3	chr7	150696111	T	G	exonic	nonsynonymous SNV	0.8	0.8732	rs1799983	hom:0,52	hom:0,49	het:44,43	hom:0,63	hom:0,57	het:30,32	hom:0,64	hom:0,52	2	14	203	1397	1
ZNF365	chr10	64415184	A	G	exonic	nonsynonymo	0.85	0.9115	rs7076156	hom:0,59	hom:0,40	hom:0,58	hom:0,41	hom:0,45	hom:0,48	hom:0,51	het:26,27	1	15	142	1458	1

						us SNV																
DNMT1	chr19	10265333	A	G	exonic	synonymous SNV		-		het:18,19	-	-	-	-	-	-	-	15	1	1600	0	0.00990099
DNMT1	chr19	10265372	C	T	exonic	synonymous SNV	0.0005	-	rs140376680	-	-	-	-	het:15,16	-	-	-	15	1	1600	0	0.00990099
PPARG	chr3	12475632	G	A	exonic	synonymous SNV		-		-	-	-	-	het:24,15	-	-	-	15	1	1600	0	0.00990099
CES1	chr16	55844509	T	C	exonic	nonsynonymous SNV		-		-	-	-	-	het:91,60	-	-	-	15	1	1600	0	0.00990099
TLR4	chr9	120470894	C	G	exonic	synonymous SNV		-		-	-	het:56,52	-	-	-	-	-	15	1	1600	0	0.00990099
